# Second-trimester abortion attitudes and practices among maternal-fetal medicine and family planning subspecialists

**DOI:** 10.1186/s12905-020-0889-9

**Published:** 2020-02-03

**Authors:** J. L. Kerns, J. K. Turk, C. M. Corbetta-Rastelli, M. G. Rosenstein, A. B. Caughey, J. E. Steinauer

**Affiliations:** 1 Department of Obstetrics, Gynecology and Reproductive Sciences, University of California, 1001 Potrero Avenue, Ward 6D, San Francisco, CA 94110 USA; 2 Department of Obstetrics, Gynecology and Reproductive Sciences, University of California, 550 16th Street, San Francisco, CA 94158 USA; 3 Department of Obstetrics and Gynecology of Oregon Health & Science University, 3181 SW Sam Jackson Park Rd, Portland, OR 97239 USA

**Keywords:** Abortion, Dilation and evacuation, Family planning, Induction termination, Maternal fetal medicine, Provider attitudes

## Abstract

**Background:**

Patients deciding to undergo dilation and evacuation (D&E) or induction abortion for fetal anomalies or complications may be greatly influenced by the counseling they receive. We sought to compare maternal-fetal medicine (MFM) and family planning (FP) physicians’ attitudes and practice patterns around second-trimester abortion for abnormal pregnancies.

**Methods:**

We surveyed members of the Society for Maternal-Fetal Medicine and Family Planning subspecialists in 2010–2011 regarding provider recommendations between D&E or induction termination for various case scenarios. We assessed provider beliefs about patient preferences and method safety regarding D&E or induction for various indications. We compared responses by specialty using descriptive statistics and conducted unadjusted and adjusted analyses of factors associated with recommending a D&E.

**Results:**

Seven hundred ninety-four (35%) physicians completed the survey (689 MFMs, 105 FPs). We found that FPs had 3.9 to 5.5 times higher odds of recommending D&E for all case scenarios (e.g. 80% of FPs and 41% of MFMs recommended D&E for trisomy 21). MFMs with exposure to family planning had greater odds of recommending D&E for all case scenarios (*p* < 0.01 for all). MFMs were less likely than FPs to believe that patients prefer D&E and less likely to feel that D&E was a safer method for different indications.

**Conclusion:**

Recommendations for D&E or induction vary significantly depending on the type of physician providing the counseling. The decision to undergo D&E or induction is one of clinical equipoise, and physicians should provide unbiased counseling. Further work is needed to understand optimal approaches to shared decision making for this clinical decision.

## Background

Women deciding to terminate a pregnancy in the second trimester for fetal anomalies or pregnancy complications can undergo one of two procedures – either a dilation and evacuation (D&E), or an induction termination. D&Es are faster, and most are done in the outpatient setting with sedation, while induction terminations typically are done on labor and delivery units, require more time, and offer more contact with the fetus [1]. Although D&E and induction termination are both safe and effective [2], the decision to undergo D&E or induction is not always driven by choice. Access to both methods varies across the United States (US) with state, local, and institutional restrictions, insurance concerns, and provider availability posing barriers, most of which disproportionately affect D&E access. Only half of all Ob/Gyn residency programs offer training in D&E [3]; fellowship training in family planning (FP) always includes D&E training, and about one-third of maternal-fetal medicine (MFM) fellowships include D&E training [4]. Beyond training, access to D&E abortions remains quite limited across the US [5]. Despite these barriers, D&E is the most common method of second-trimester abortion [6] and women seeking abortion for fetal anomalies are still more likely to undergo D&E [7].

Studies have shown that provider opinion and preference affect health-related communication and clinical decisions [8, 9] and can be a barrier to high-quality care [10]. Because abortion skills are taught differentially according to subspecialty and geographic area of the US, there is good reason to believe that Ob/Gyn physicians, and specifically those who counsel women seeking second-trimester abortion, have different preferences for methods of second-trimester abortion – that is, D&E or induction termination.

Women value the ability to choose their method of abortion [11] but are not always offered both methods [7]. Women deciding between D&E or induction termination frequently interact with either MFM or FP physicians, and the counseling they receive may differ based on physician subspecialty. We sought to compare MFM and FP physicians’ attitudes and practices around second-trimester abortion for abnormal pregnancies. The findings from our study will inform any future strategies to improve patient-centered and unbiased counseling for patients deciding between D&E and induction termination.

## Methods

We conducted an anonymous survey in 2010–2011 of all US members of the Society for Maternal-Fetal Medicine (SMFM) and FP subspecialists, defined as all faculty and fellows associated with the Family Planning Fellowship (FFP). SMFM provided names and postal addresses for SMFM members. We obtained names and email addresses of current and former FP fellows from the national FFP office. In addition, the directors at each fellowship site provided the names and emails of FP faculty. We obtained email addresses for some MFMs from publicly available information, such as publications on PubMed or institutional websites. We invited all subjects for whom we had an email address to complete an online survey using Key Survey. In accordance with human subjects approval, participants’ informed consent was provided by participants launching the survey. Several analyses have been published from the parent survey on D&E and inductions practices among family planning and maternal-fetal medicine subspecialists [4, 12, 13].

The survey probed respondents for their beliefs about patient preferences and their understanding of safety regarding D&E or induction for various indications, as well as their recommendations between D&E or induction termination for different case scenarios. In order to understand their opinions about patient preference and method safety, we presented five indications for termination: undesired pregnancy, fetal anomaly, severe maternal morbidity, abortion between 14 and 19 weeks, and abortion between 20 and 24 weeks. We asked physicians which method (D&E or induction or no preference) patients generally prefer for the above indications and which method physicians thought was safer (D&E or induction or no difference in safety). We also presented case scenarios for a patient requesting abortion at 20 weeks’ gestation for six different reasons: trisomy 21, renal agenesis, intrauterine fetal demise (IUFD), severe pre-eclampsia, chorioamnionitis with sepsis, and preterm premature rupture of membranes (PPROM). We asked physicians which method they would recommend for each scenario (recommend D&E, recommend D&E but patient’s choice, no recommendation, recommend induction but patient’s choice, or recommend induction). We asked physicians which method is most commonly done at their institution for these scenarios.

We collected demographic information, practice characteristics, training in and provision of second-trimester abortion and institutional regulations and barriers to offering second-trimester abortion services. To ensure anonymity, respondents were only asked to identify the region of the US and population size of the city where they practice. We assessed intrinsic religious motivation [14] using three validated questions with true or false responses; scores ranged from 0 to 3 with higher scores reflecting greater intrinsic religious motivation [14]. We assessed abortion attitudes using a validated scale with five questions on a five-point Likert scale [15]. Scores ranged from 5 to 25, with higher scores reflecting more favorable abortion attitudes. We defined FP exposure as presence of an FP fellowship at a past or current institution.

We compared MFM and FP attitudes towards, and recommendations for, D&E or induction termination. We used descriptive statistics to compare responses by specialty for indications and case scenarios. We analyzed the recommendation responses as “any recommendation”, “patient choice”, or “no recommendation”. We conducted unadjusted and adjusted analyses of factors associated with recommending a D&E for each case scenario. For the adjusted analysis, we dichotomized responses into D&E recommendation (“recommend D&E” and “recommend D&E, but patient’s choice”) versus induction or no recommendation (“no recommendation”, “recommend induction” and “recommend induction, but patient’s choice”). We also did an adjusted analysis of factors associated with MFM recommendations for D&E. We determined a priori to include age, gender, practice setting, religious and abortion attitudes, and providing D&Es as covariates. Exposure to an FP fellowship (past or current) was only included in the analysis with MFM physicians. We offered respondents a $5 gift card that was not contingent upon starting or completing the survey. We performed all analyses using Stata 12 (StataCorp 2011, College Station, TX). The University of California, San Francisco Committee on Human Research approved this study.

## Results

Of the 2294 subjects (2125 MFMs, 169 FPs) who received an email or paper survey invitation, 794 (35%) responded (689 MFMs, 105 FPs). Most participants were women under 50 years of age. Most worked in an academic setting (71%) and were fellowship trained (98%). About one in five MFM respondents had exposure to a FP fellowship (Table 1).

**Table 1:** Characteristics of maternal-fetal medicine (MFM) and family planning (FP) subspecialists who responded to the survey

Characteristic	Total N (%)	MFM n (%)	FP n (%)
Total	794	689 (87)	105 (13)
Demographics			
Age, years^a,b^		47 ± 10	39 ± 8
Female^b^	442	352 (51)	90 (86)
West region^b^	206	174 (25)	32 (30)
Northeast region^b^	257	222 (32)	35 (33)
South/Southeast region^b^	168	158 (23)	10 (10)
Midwest region^b^	157	130 (19)	27 (26)
Home city population < 1 million^b^	446	394 (57)	52 (50)
Clinical Practice			
Supervises trainees (fellows, residents)	700	599 (87)	101 (96)
Works > 50% time in academic setting^b^	563	470 (68)	93 (89)
Fellowship trained	775	685 (99)	90 (86)
Family planning fellowship at current institution^b^	229	143 (21)	86 (82)
Family planning fellowship at previous institution^b^	181	150 (22)	31 (30)
Provide D&Es	324	224 (33)	100 (95)
Personal Beliefs			
Intrinsic religious motivation^a,b^		2.3 ± 0.9	2.6 ± 0.7
Abortion attitudes^a,b^		17 ± 4	22 ± 3

^a^mean ± SD, not n (%)

^b^missing data

Recommendations for D&E were more common than actual D&E provision at respondents’ institutions. Among all respondents (MFMs and FPs combined), D&E was recommended with the following frequency: 47% for trisomy 21, 42% for renal agenesis, 35% for IUFD, 49% for severe pre-eclampsia, 46% for chorioamnionitis with sepsis, and 26% for PPROM. D&E was the most common method done at their institution with the following frequency: 49% for trisomy 21; 37% for renal agenesis; 24% for IUFD; 26% for severe pre-eclampsia; 29% for chorioamnionitis with sepsis; 14% for PPROM.

When presented with six case scenarios and asked about how they would counsel women for D&E or induction, MFMs were roughly twice as likely to make any recommendation (either for D&E or induction) compared to FPs for the following scenarios (10% vs 5% trisomy 21; 15% vs 7% renal agenesis; 18% vs 10% IUFD; 22% vs 7% PPROM) (Table 2). For cases of maternal disease, MFMs and FPs were approximately as likely to make a recommendation (32% vs 28% severe pre-eclampsia; 49% vs 55% chorioamnionitis with sepsis). For all scenarios other than chorioamnionitis, MFMs were less likely than FPs to state that recommendations should be driven by patient choice (54% vs 76% trisomy 21; 57% vs 72% renal agenesis; 58% vs 60% IUFD; 49% vs 61% severe pre-eclampsia; 45% vs 63% PPROM).

**Table 2:** Proportion of maternal-fetal medicine (MFM) and family planning (FP) subspecialists who gave a recommendation for D&E or induction only, a recommendation for D&E or induction but patient’s choice, or gave no recommendation for each case scenario

	Total	MFM N (%)	FP N (%)
*TOTAL*		689	105
*Trisomy 21*			
Recommendation (D&E or induction)	77	72 (10)	5 (5)
D&E	46	41 (6)	5 (5)
Induction	31	31 (4)	0 (0)
Patient choice (D&E or induction)	450	370 (54)	80 (76)
D&E	322	243 (35)	79 (75)
Induction	128	127 (18)	1 (1)
No recommendation	263	243 (35)	20 (19)
*Renal agenesis*			
Recommendation (D&E or induction)	109	102 (15)	7 (7)
D&E	63	56 (8)	7 (7)
Induction	46	46 (7)	0 (0)
Patient choice (D&E or induction)	471	395 (57)	76 (72)
D&E	270	195 (28)	75 (71)
Induction	201	200 (29)	1 (1)
No recommendation	211	189 (27)	22 (21)
*Intrauterine fetal demise*			
Recommendation (D&E or induction)	135	125 (18)	10 (10)
D&E	54	46 (7)	8 (8)
Induction	81	79 (11)	2 (2)
Patient choice (D&E or induction)	473	402 (58)	71 (68)
D&E	225	157 (23)	68 (65)
Induction	248	245 (36)	3 (3)
No recommendation	180	156 (23)	24 (23)
*Severe pre-eclampsia*			
Recommendation (D&E or induction)	252	223 (32)	29 (28)
D&E	148	119 (17)	29 (28)
Induction	104	104 (15)	0 (0)
Patient choice (D&E or induction)	403	339 (49)	64 (61)
D&E	242	180 (26)	62 (59)
Induction	161	159 (23)	2 (2)
No recommendation	135	123 (18)	12 (11)
*Chorioamnionitis with sepsis*			
Recommendation (D&E or induction)	398	340 (49)	58 (55)
D&E	207	153 (22)	54 (51)
Induction	191	187 (27)	4 (4)
Patient choice (D&E or induction)	313	275 (40)	38 (36)
D&E	156	122 (18)	34 (32)
Induction	157	153 (22)	4 (4)
No recommendation	80	71 (10)	9 (9)
*Preterm premature rupture of membranes*			
Recommendation (D&E or induction)	161	154 (22)	7 (7)
D&E	45	38 (6)	7 (7)
Induction	116	116 (17)	0 (0)
Patient choice (D&E or induction)	379	313 (45)	66 (63)
D&E	161	99 (14)	62 (59)
Induction	218	214 (31)	4 (4)
No recommendation	250	219 (32)	31 (30)

**Fig. 1:**
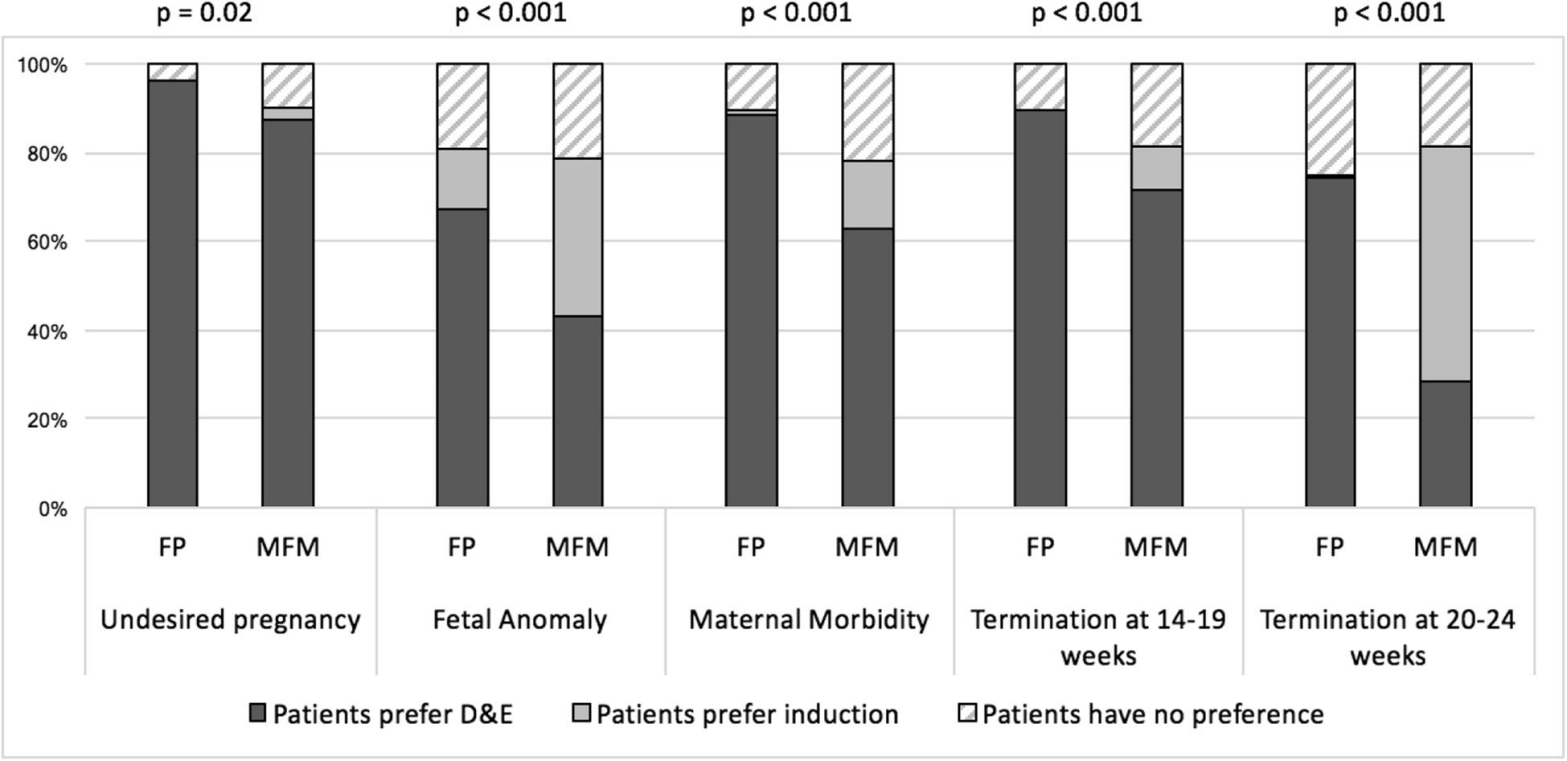
Proportions of FP and MFM physicians who believe patients prefer D&E, induction, or have no preference for different indications

**Fig. 2:**
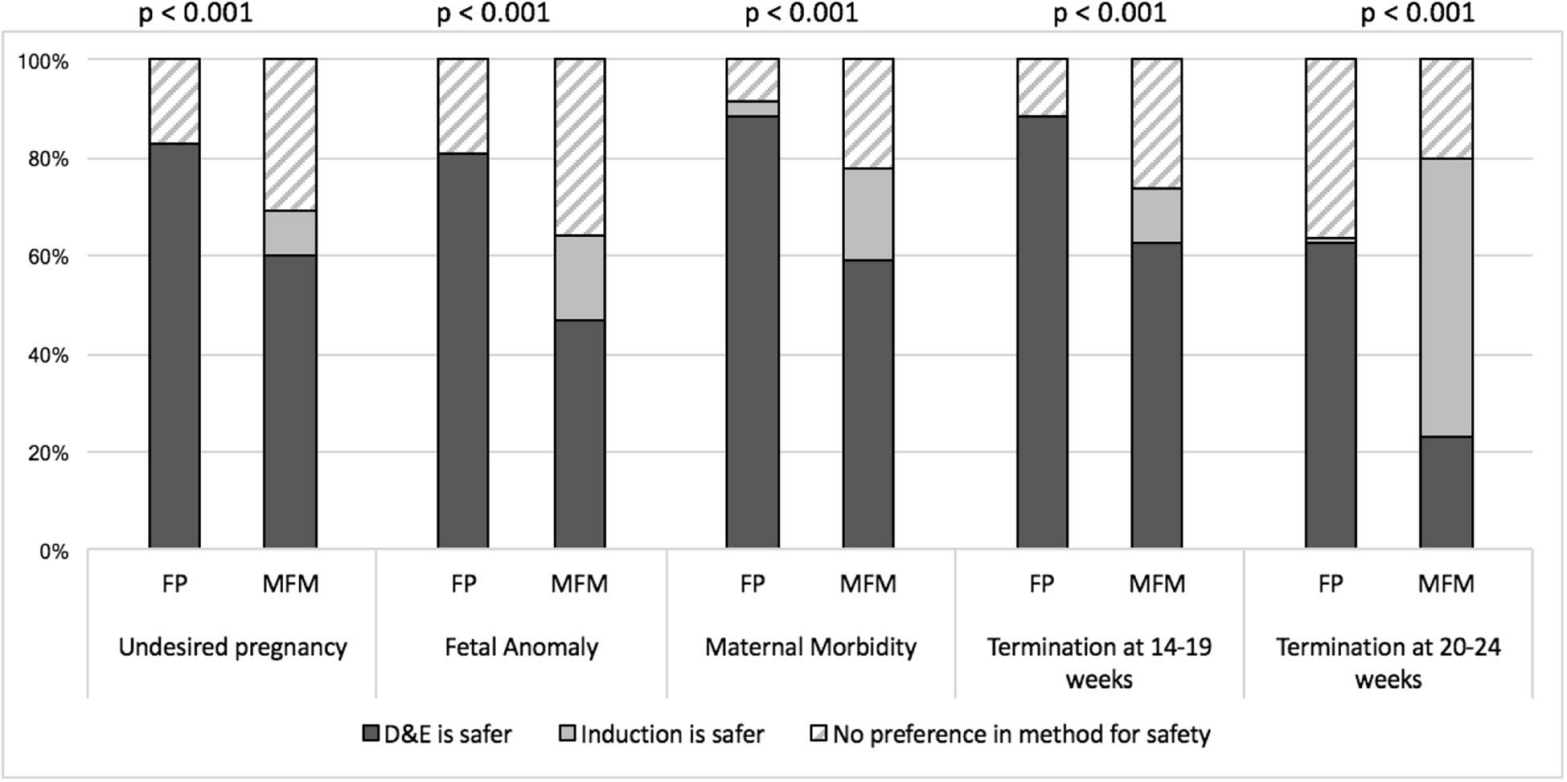
Proportions of FP and MFM physicians who believe D&E is safer, induction is safer, or no difference in safety for different indications

MFMs were less likely than FPs to believe that patients prefer D&E (Fig. 1) for undesired pregnancies, fetal anomalies, maternal morbidity, abortions between 14 and 19 weeks and abortions between 20 and 24 weeks (*p* ≤ 0.02 for all). MFMs were less likely than FPs to feel that D&E was a safer method (compared to induction or neither) for all of the same indications listed above (Fig. 2) (*p* < 0.01 for all).

In unadjusted analyses, there was a statistically significant difference between MFM and FP recommendations for all case scenarios (Table 3). When asked whether they would recommend D&E for a patient requesting termination at 20 weeks’ gestation for specific scenarios, MFMs were less likely than FPs to recommend D&E for trisomy 21 (41% vs 80%, respectively); renal agenesis (37% vs 78%, respectively); IUFD (30% vs 72%, respectively); severe pre-eclampsia (44% vs 87%, respectively); chorioamnionitis with sepsis (40% vs 84%, respectively); PPROM (20% vs 66%, respectively) (*p* < 0.001 for all).

**Table 3:** Unadjusted and adjusted odds of a family planning (FP) versus maternal-fetal medicine (MFM) physician recommending D&E for each case scenario

Case scenario	FP n (%)	MFM n (%)	Unadjusted OR (95% CI)	Adjusted^a^ OR (95% CI)
Trisomy 21				
Induction recommendation or no recommendation^b^	21 (20)	401 (59)	–	–
D&E recommendation^c^	84 (80)	284 (41)	5.7 (3.4–9.3)	3.9 (2.1–7.0)
Renal agenesis				
Induction recommendation or no recommendation	23 (22)	435 (63)	–	–
D&E recommendation	82 (78)	251 (37)	6.2 (3.8–10.1)	4.3 (2.4–7.6)
Intrauterine fetal demise				
Induction recommendation or no recommendation	29 (28)	480 (70)	–	–
D&E recommendation	76 (72)	203 (30)	6.2 (3.9–9.8)	4.2 (2.4–7.3)
Severe pre-eclampsia				
Induction recommendation or no recommendation	14 (13)	386 (56)	–	–
D&E recommendation	91 (87)	299 (44)	8.4 (4.7–15.0)	4.6 (2.4–8.8)
Chorioamnionitis with sepsis				
Induction recommendation or no recommendation	17 (16)	411 (60)	–	–
D&E recommendation	88 (84)	275 (40)	7.7 (4.5–13.3)	4.2 (2.2–7.8)
Preterm premature rupture of membranes				
Induction recommendation or no recommendation	35 (34)	549 (80)	–	–
D&E recommendation	69 (66)	137 (20)	7.9 (5.0–12.4)	5.5 (3.1–9.7)

^a^Adjusted for age, gender, practice setting, religiosity, abortion attitude, provide D&Es

^b^Induction recommendation or no recommendation includes responses “no recommendation”, “recommend induction”, and “recommend induction, but patient’s choice”

^c^D&E recommendation includes responses “recommend D&E” and “recommend D&E, but patient’s choice”

In our adjusted analyses of FPs and MFMs (Table 3), we found that FPs had 3.9 to 5.5 times higher odds of recommending D&E for all case scenarios. Higher physician age was significantly associated with a D&E recommendation for trisomy 21, renal agenesis, IUFD, and PPROM (*p* ≤ 0.05 for all). Greater religiosity was significantly associated with a D&E recommendation for trisomy 21, renal agenesis, IUFD and PPROM (*p* ≤ 0.05 for all). Working in a non-academic setting was associated with lower odds of recommending D&E for chorioamnionitis (*p* = 0.001) and providing D&Es was associated with higher odds of recommending D&E for chorioamnionitis (*p* = 0.01). A more favorable abortion attitude and being a FP provider were independent predictors of recommending D&E for all case scenarios (*p* < 0.01 for all).

In our adjusted analyses of MFMs only, we found that MFMs with exposure to family planning had greater odds of recommending D&E for all case scenarios compared to MFMs without exposure to family planning (Table 4). Higher physician age was significantly associated with a D&E recommendation for trisomy 21, renal agenesis, IUFD, and PPROM. Greater religiosity was significantly associated with a D&E recommendation for renal agenesis, IUFD, chorioamnionitis, and PPROM. More favorable abortion attitudes were significantly associated with a D&E recommendation for all case scenarios, except IUFD. Providing D&Es increased the odds of recommending D&E for chorioamnionitis. Additionally, we found lower odds of recommending D&E for chorioamnionitis by physicians working in a non-academic setting.

**Table 4:**
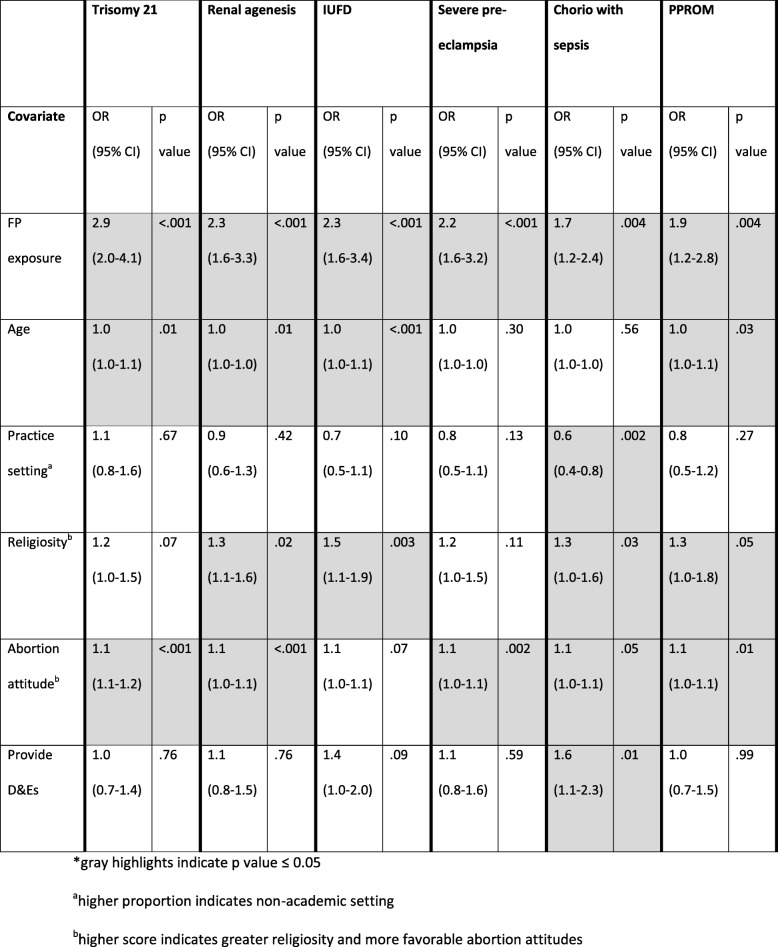
Association of family planning (FP) exposure, past or current, with D&E recommendation by maternal-fetal medicine (MFM) physicians for each case scenario

*gray highlights indicate *p* value ≤0.05

^a^higher proportion indicates non-academic setting

^b^higher score indicates greater religiosity and more favorable abortion attitudes

## Discussion

In this study we investigated MFM and FP physicians’ attitudes and practices around second-trimester abortions for abnormal pregnancies. MFMs were less likely than FPs to believe that patients prefer D&E and to make a recommendation for D&E for all case scenarios. MFMs with exposure to family planning were more likely to recommend D&E compared to MFMs without exposure.

Our findings are consistent with other studies showing that physicians’ biases affect recommendations for treatments around pregnancy termination. In one survey of MFM physicians, counseling around management of life-threatening fetal anomalies differed according to physician demographics, with younger physicians and those in academic practice more likely to offer a choice of either induction or D&E [16]. One study of MFMs found that physicians’ negative attitudes towards late abortion and physicians practicing outside of the Western region were associated with a lower likelihood of discussing termination as an option [17]. We found religiosity to be associated with physicians’ recommendations for D&E or induction, a finding consistent with a study demonstrating that religious primary care physicians give less weight to a patient’s expressed wishes and values when making an ethically complex medical decision [18]. Counseling recommendations that patients receive for second-trimester abortion can vary widely based on the individual physician.

We found striking differences around physicians’ beliefs and attitudes about safety for both methods (D&E versus induction), with MFMs more likely than FPs to believe that D&E is less safe than induction across all queried indications. With respect to major complications, such as hemorrhage, infection or additional major surgery, D&E is at least as safe, if not safer, than induction termination [2, 19]. The American College of Obstetricians and Gynecologists advises physicians to offer patients a choice between methods given that there is clinical equipoise with regard to the safety of each procedure [19]. And research demonstrates that patients highly value having a choice [7, 11]; however, our findings indicate that there is a significant difference between MFM and FP physicians’ beliefs about what method patients prefer. This likely reflects a cultural difference between MFMs and FPs, and our respondents’ beliefs about patient preferences may, in fact, have more to do with their preferences rather than patient preferences. Physicians who wish to authentically engage in shared decision making and support patients in values-driven decisions for abortion might consider reflective tools [20] to help clarify the effects of their personal beliefs on recommendations around D&E or induction.

We observed that MFM physicians with exposure to family planning (either current or past) were more likely to recommend D&E than MFMs without exposure. We know that training in abortion provision increases residents’ intention to provide abortion services after residency [21], with even partial participation being associated with increased acceptance of abortion [22]. Increasing exposure to abortion care and training also improves trainees’ experiences and skill sets. Three-quarters of MFM fellows believe that D&E training should be included during fellowship [12]; however, many MFMs will encounter barriers to providing abortions [13]. By strengthening collaborations between FP and MFM fellowships, we can provide more training opportunities for MFM fellows. In addition, building viable relationships with nearby FP fellowships or clinics can improve referral networks and patient-centered care.

Given the variety of recommendations patients receive around pregnancy termination, a standardized approach should be taken to adequately inform and equip women regarding their options. Shared-decision making is a counseling strategy that is effective in increasing decisions consistent with patients’ values [23, 24]. By engaging in shared-decision making and standardizing recommendations around pregnancy termination, patients will receive more comprehensive and personalized care around this sensitive decision.

Increasing collaboration and strengthening referral networks between MFM and FP programs may provide both subspecialists with improved expertise and counseling strategies around second-trimester abortions for abnormal pregnancies. Health policy makers have repeatedly called for implementation of interdisciplinary collaboration as a key approach to improve the quality and safety of patient care [25]. The use of interprofessional activities with an external facilitator or interprofessional meetings were found to improve adherence to recommended practices [26]. By creating opportunities for MFM and FP subspecialists to work together, patients will receive more inclusive clinical care.

There are limitations to our study. First, as a cross-sectional study, we are only able to determine associations but not causality. Second, although our response rate is comparable to previous surveys [16, 17], it is unclear whether our sample is representative of all MFM and FP physicians. We also recognize that there are many reasons why physicians may recommend a D&E or induction, most notably logistical barriers to D&E. While we did not assess reasons for a D&E or induction recommendation with each case scenario, we did assess physicians’ beliefs about the relative safety and patient preferences for each method as a way to contextualize the responses to the case scenarios.

## Conclusions

MFM and FP physicians commonly counsel patients around method of abortion procedure in the setting of fetal anomalies or pregnancy complications, and their biases likely affect the recommendation that patients receive. Counseling around D&E or induction should be approached from a shared decision-making framework. Such an approach would minimize provider bias, maximize a values-based decision, and improve patient-centered care.

## Data Availability

The datasets used and/or analyzed during the current study are available from the corresponding author on reasonable request.

## Abbreviations

D&E: Dilation and evacuation
FP: Family planning
MFM: Maternal-fetal medicine
